# Volume-based bias in automated measurements of lateral ventricle and hippocampal volumes of mild traumatic brain injury patients

**DOI:** 10.1016/j.ynirp.2026.100361

**Published:** 2026-06-12

**Authors:** Lauren Carter, Tapasya Surti, Tracy J. Abildskov, Eddie Shum, Amanda R. Hertzler, Meghana Dodda, Alan Rodriguez, Colin Brown, Justin Roy Sanchez, Deen Tar, Leon Ma, Sean Beaty, Enrico Fazzini, Cheryl E. Hightower, Travis H. Snyder

**Affiliations:** a Imgen Research Group, Imgen LLC., Las Vegas, NV, USA; b College of Osteopathic Medicine, Touro University Nevada, Henderson, NV, USA; c Department of Neurology, University of Texas Health Science Center, Houston, TX, USA; d Department of Neurology, University of Utah, Salt Lake City, UT, USA; e Department of Neurological Surgery, University of Pittsburgh, Pittsburgh, PA, USA; f Department of Neurology, Riverside University Health System, Moreno Valley, CA, USA; g Department of Anesthesiology, Loyola University Medical Center, Maywood, IL, USA; h HCA Healthcare, Nashville, TN, USA; i Mountain View Hospital, Las Vegas, NV, USA; j SimonMed, 16220 N. Scottsdale Rd., Suite 600, Scottsdale, AZ, USA

**Keywords:** Automated segmentation, Lateral ventricle, Hippocampus, Manual segmentation, Reliability, Structural MRI

## Abstract

Lateral ventricle and hippocampal volume are clinical indicators for many disorders. Manual tracing is time-consuming; the alternative, automated tracing, may be inconsistent across software programs. The purpose was to estimate the reliability of three automated measuring programs relative to manual tracing, and to consider the use of linear inter-conversion factors. Participants with mild traumatic brain injury (mTBI) were measured for lateral ventricle volume (LVV; n = 20; 35% male; mean age = 36.5) or hippocampal volume (HV; n = 50; 42% male; mean age = 39.1). Axial 4 mm T2 images and 1.2 mm coronal T1 Images were used for manual lateral ventricle tracings and hippocampal tracings, respectively (InteleViewer®). 3T MR images 1.2 mm sagittal T1 sequences were used for automated volumetric tracings in NeuroQuant® v3.0, FreeSurfer® v6, and volBrain software. Reliability was estimated by intraclass correlation coefficients (ICCs), and relationships between software programs were described using regression equations. Human inter-rater reliability was excellent (ICC = 0.99) for LVV and moderate (0.67) for HV. For LVV, software programs had excellent consistency with manual tracing and with each other (≥0.97). There was excellent agreement between manual tracing and NeuroQuant® (0.97), but moderate-good agreement (0.54-0.87) with FreeSurfer® or VolBrain, with notably size-biased underestimation of LVV. For HV, FreeSurfer® and VolBrain had excellent consistency with manual tracing (≥0.90); NeuroQuant® had moderate-good consistency with other methods (0.57-0.79). FreeSurfer® had excellent agreement with manual tracing (0.91), but poor-good agreement with other software (0.42-0.78). Compared to manual tracing, FreeSurfer® overestimated large HV, and volBrain underestimated small HV. Conversions involving HV may be ill-advised given their low consistency. Algorithms for brain volumetrics must be evaluated for distorting effects of size-associated bias.

## Highlights

• Accuracy of automated measurements varies according to region.
• Automated measurements have size-associated biases unique to program and region.
• Volumes for regions with well-defined borders can more reliably be converted among tracing methods.

## 1 Introduction

Assessment of the size of brain structures has always been an important component of magnetic resonance imaging (MRI)-based neuroradiological evaluation, with determinations made qualitatively based on the experience and training of the interpreting physician. Historically, qualitative volumetric measurements were made by manually tracing the outline of a structure on serial uniplanar imaging using standard picture archiving and communication systems (PACS), such as InteleViewer® (InteleRad; https://www.intelerad.com/) or OsiriX (Rosset et al., 2004). In such systems, embedded tools facilitate the manual tracing on each slice and, provided there is some overlap in the serial area measurements in the third plane, volumes are summed from the areas using slice thickness to obtain a total 3D volume in mm^3^ for the traced region of interest (ROI). The time-consuming nature of manual tracing is prohibitively expensive and impractical on a large scale in clinical practice (Schmidt et al., 2018). Various automated methods have been developed to quickly and automatically calculate region of interest (ROI) total volume, but widespread use of automated methods requires clear understanding of reliability, validity, and the distribution of values relative to manual tracing. Since their inception, automated methods have been a frequent subject of research studies (Hedges et al., 2022; Ochs et al., 2015; Ross et al., 2013; Schoemaker et al., 2016), some of which have theorized that automated segmentation of certain structures, particularly for those which are inherently challenging to recognize (e.g., those with irregular invaginations), may eventually trump traditional visual inspection by a radiologist (Ochs et al., 2015; Ross et al., 2013). Currently, different automated approaches are favored by different user groups: researchers are more likely to use FreeSurfer®, whereas FDA-cleared software such as NeuroQuant® is more used by clinicians (Ross et al., 2018). Approaches are being developed using machine learning or deep learning algorithms to accomplish this task as well.

Automated methods have distinct approaches to segmenting ROI in the brain. FreeSurfer® segmentation uses a nonstationary anisotropic Markov Random Field to assign each voxel in an image to a ROI, a method which allows for neighboring voxels to be inconsistently related to each other and incorporates information about relative ROI positions (Fischl et al., 2002). NeuroQuant® segmentation started with the same basic approach as FreeSurfer®, but further details appear to be proprietary. The package volBrain also uses an initial voxel assignment, followed by a process to refine the initial classifications (Manjon and Coupe, 2016) and segment using non-local multi-atlas label fusion. In the final segmentation step, the patch size is allowed to vary to account for different ROI sizes and shapes.

**Fig. 1:**
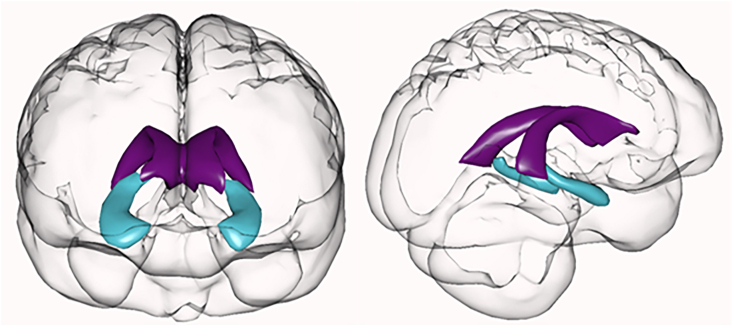
Anterior (left panel) and lateral (right panel) views of the brain with hippocampus in blue and the lateral ventricle in purple. Created in (Bakker et al., 2015).

The focus of this study is on the comparative reliability (precision and accuracy) and distribution of values resulting from automated segmentation of two ROI in the brain, selected for their notable clinical relevance: the lateral ventricles and the hippocampus (Fig. 1). Although the two are grossly similar in morphology and complexity of shape, the margins of the ventricles are clearly delineated, while the curved and looping hippocampal margins are more challenging.

The lateral ventricles are centrally located, paired, fluid filled cavities in the cerebrum, with one in each hemisphere. The lateral ventricles contain the vast majority of the CSF (7-10 ml per hemisphere) associated with the ventricular system (Wright et al., 2012). Their volume is dictated by the surrounding structures, such as the corpus callosum, thalamus, caudate nucleus, septum pellucidum, and fornix. Since the lateral ventricle volume (LVV) is related to the volume of multiple ROI, expansion is indicative of brain tissue atrophy. LVV is related to a variety of neurological medical disorders and conditions. Conditions that have been linked to enlargement of LVV include malignant hypertension (Salerno et al., 1992), diabetes (Currà et al., 2024; Jacobs, 1977), Alzheimer's disease (Förstl et al., 1995) and other neurodegenerative conditions (Pirttilä et al., 1993), psychological conditions such as schizophrenia (Kempton et al., 2011), traumatic brain injury (TBI) (Anderson and Bigler, 1995) and carbon monoxide poisoning (Gale et al., 1999).

The hippocampus, reviewed in (Anand and Dhikav, 2012), is a C-shaped structure in the temporal lobe largely enveloped by the temporal horn of the lateral ventricle. Although the boundary with the lateral ventricle is easily identified due to brain/fluid interface, and the posterosuperior thinner articulation with the stalk like fornix is typically distinguishable, the boundary between the hippocampus and its anterior and inferomedial border with the amygdala, parahippocampal gyrus, and entorhinal cortex offer a more significant challenge for segmentation algorithms (Matsuoka et al., 2003; Schroder and Pantel, 2016). Hippocampal atrophy is among the most well-studied brain regions for disparate pathologic conditions, including Alzheimer's disease (Schuff et al., 2009), mild traumatic brain injury (Monti et al., 2013; Zagorchev et al., 2016), hypoxic ischemic encephalopathy (Pfister et al., 2023), and carbon monoxide poisoning (Chu et al., 2004).

Because of their strong associations with these pathological processes and demonstrated clinical relevance, underestimating or overestimating LVV or HV could result in erroneous or delayed diagnoses, unnecessary follow-up testing, or inaccurate understanding of disease progression. Volumetric information is most useful to clinicians when it is provided as z-scores or percentiles adjusted for age and intracranial volume, but doing so requires a population mean and standard deviation that is relevant to the patient and accurate image collection and processing. Serial imaging results, particularly if there are prior scans or if the initial MRI is performed in or proximal to the acute phase of an insult can be useful to clinicians, but images must be directly comparable among time points. Due to the existence of multiple software programs for volumetric assessment, it would be a helpful clinical and research tool to confidently combine volumetric measurements that come from different software programs into a single unified dataset.

The purpose of this study was to characterize reliability and measurement biases of three automated software programs relative to the hand-drawn gold standard of a radiologist for measuring LVV and HV in participants diagnosed with mTBI. A secondary goal was to investigate the use of equations to convert between software programs. Although not precisely the same, the purpose and scope of this study is similar to that of Ross et al. (2018), who measured 20 patients with mTBI (mean age = 44), 20 healthy controls (mean age = 68), and 20 patients with Alzheimer's Disease (AD; mean age = 68), with the latter two groups from the Alzheimer's Disease Neuroimaging Initiative (ADNI) database (adni.loni.ucla.edu).

## 2 Methods

This was a retrospective study based on existing imaging and deidentified patient information. All participants had been diagnosed with mild traumatic brain injury (mTBI) by a board-certified neurologist specializing in head trauma based on standard DSM-V diagnostic criteria (APA, 2013). Patients were randomly sampled using numbers provided by random.org from a larger dataset of 250 people after excluding potential participants if they had abnormal MRI findings or conditions (other than sex or age) that are known to affect MRI volumetric measurements, as fully described in (Vanier et al., 2020). The study was determined exempt by the Touro University Nevada Institutional Review Board (IRB4-5-17D).

Participants were imaged using a 3.0 T MR Signa HDxt system (G.E. Healthcare Milwaukee WI) equipped with an 8-channel head coil using a brain MRI protocol. Standard sequences included Axial T2 imaging with in-plane resolutions of 0.45 mm and a slice thickness of 4.0 mm. Lateral ventricles and hippocampus boundaries were manually traced on Axial T2 images and Coronal 1.2 mm T1 images respectively using InteleViewer®, which was used for this study because it was integrated into our existing PACS. A screenshot showing manual tracing of the hippocampus in one slice is shown in Fig. S1. The boundaries of the hippocampus were known a priori to be more difficult to trace, so human tracing reliability was based on two raters for LVV and four raters for HV, and the sample size for HV was larger (20 for LVV compared to 50 for HV) in anticipation of more uncertainty. The mean volume from the raters in InteleViewer® was considered the most accurate ‘reference’ volume in the analysis. A sagittal 3D T1-weighted sequence with a voxel size of 0.93 x 0.93 × 1.2 mm (TE 2.208ms, TR 5.396ms) was used to identify and estimate LVV and HV using the three aforementioned automated volumetric software programs: NeuroQuant® v3.0, FreeSurfer® v6, and volBrain.

Reliability of volume measurements across software programs was assessed using the intraclass correlation coefficient (ICC; type (3,k) (Shrout and Fleiss, 1979; McGraw and Wong, 1996);) based on a single-rating, 2-way mixed effects model. Reliabilities were estimated for consistency and agreement. Measurements have high consistency when they can be converted between programs by a simple conversion, even though the mean volume may differ between programs. Measurements have high agreement if the numeric values across programs are the same. Low consistency and low agreement suggest that numeric values cannot be converted across programs. Estimated ICCs are reported with 95% confidence intervals.

To gain more insight into the relative volumes estimated by different automated segmentation methods, offset biases and the linear or non-linear volume-based biases across methods were also considered on a pairwise basis between software programs. If conversion is reasonable based on consistency, an offset bias is indicated when the relationship between two programs has a linear slope of one, but the intercept is not zero. If so, then conversion between programs requires addition or subtraction of a constant to make volume measurements equivalent between the two programs. A linear or non-linear volume-based bias occurs when the slope between volume measurements made by two programs does not have a linear slope of one, or if the relationship is non-linear. Volume-based biases require application of a volume-based function to convert from one program to another. Graphical analysis using locally weighted sum of squares (loess) lines was used to first confirm that the relationship between programs was linear. To further confirm absence of non-linearity, the simple linear model was compared to polynomial models incorporating quadratic and cubic terms using the Akaike Information Criterion (AIC). In every model, minimum AIC was observed with the linear model, supporting the use of a linear model.

Coefficients and statistics that could be used to convert from one measurement method to another were estimated using simple least-squares regressions. The 95% bias-corrected quantile confidence intervals were estimated based on 1000 bootstrap samples (Davison and Hinkley, 1997). Analyses were performed in Rv4.4.1 (R Core Team, 2024).

## 3 Results

For measuring LVV (n = 20 with 7 males), participants were 17-64 year of age (mean = 36.5), with mean female total intracranial volume (TIV) of 1458 (SD = 98) cm^3^ and mean male TIV of 1612 (SD = 57) cm^3^. Distribution of age for LVV was 1 (5%) under 18 years of age, 12 (60%) 18-40 years, 5 (25%) 41-60 years, and 2 (10%) over 60 years. For measuring HV (n = 50 with 21 males), participants were 12-77 years of age (mean = 39.1), with mean TIV of females 1415 (SD = 103) cm^3^ and mean TIV for males 1630 (SD = 125) cm^3^. Distribution of age for HV was 3 (6%) under 18 years of age, 26 (52%) 18-40 years, 15 (30%) 41-60 years, and 6 (12%) over 60 years. Inter-rater reliability among human raters using InteleViewer® was excellent for LVV (ICC = 0.99, 95% confidence interval = 0.97, 1.00) and moderate for HV (ICC = 0.67, 95% confidence interval = 0.50, 0.75).

Across measurement platforms, consistency reliabilities for LVV (Table 1) were in the excellent range (ICC≥0.97), whereas reliabilities based on agreement varied: excellent (ICC = 0.97) for InteleViewer® with NeuroQuant®, good for FreeSurfer® with InteleViewer® (ICC = 0.76), NeuroQuant® (ICC = 0.85), and volBrain (ICC = 0.87), and moderate for volBrain with InteleViewer® (ICC = 0.54) and NeuroQuant® (ICC = 0.62). For HV, reliabilities based on consistency were excellent for InteleViewer® with FreeSurfer® (ICC = 0.94) and with volBrain (ICC = 0.90), good for NeuroQuant® with InteleViewer® (ICC = 0.79) and FreeSurfer® with volBrain (ICC = 0.89), and moderate for NeuroQuant® with FreeSurfer® (ICC = 0.73) and volBrain (ICC = 0.57). Reliabilities based on agreement for HV were excellent for InteleViewer® with FreeSurfer® (ICC = 0.91), good for InteleViewer® with NeuroQuant® (ICC = 0.75) and volBrain (ICC = 0.78), moderate for FreeSurfer® with NeuroQuant® (ICC = 0.72) and volBrain (ICC = 0.68), and poor between NeuroQuant® and volBrain (ICC = 0.42).

**Table 1:** Consistency and agreement reliabilities (ICC and 95% confidence intervals) between pairs of software programs used to measure LVV and HV.

		Lateral Ventricle Volume		Hippocampal Volume	
		Consistency	Agreement	Consistency	Agreement
InteleViewer®	NQ	0.99 (0.96, 0.99)	0.97 (0.76, 0.99)	0.79 (0.66, 0.88)	0.75 (0.49, 0.87)
	FreeSurfer®	0.98 (0.94, 0.99)	0.76 (0.00, 0.95)	0.94 (0.90, 0.97)	0.91 (0.67, 0.96)
	volBrain	0.97 (0.91, 0.99)	0.54 (0.00, 0.87)	0.90 (0.83, 0.94)	0.78 (0.04, 0.93)
NQ	FreeSurfer®	0.98 (0.96, 0.99)	0.85 (0.00, 0.97)	0.73 (0.56, 0.83)	0.72 (0.56, 0.83)
	volBrain	0.97 (0.92, 0.99)	0.62 (0.00, 0.90)	0.57 (0.35, 0.73)	0.42 (0.00, 0.69)
FreeSurfer®	volBrain	1.00 (0.99, 1.00)	0.87 (0.00, 0.97)	0.89 (0.81, 0.93)	0.68 (0.00, 0.90)

**Fig. 2:**
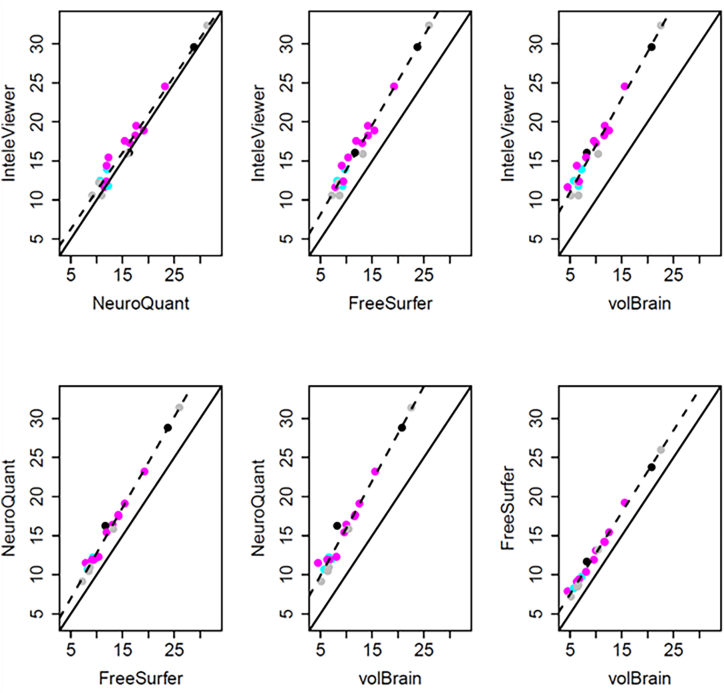
Illustrative relationships between lateral ventricle volumes (cm^3^) measured using different pairs of software programs. The best fit line is dashed (model information in Table 3, S1), and the reference line for perfect agreement is solid (slope = 1, intercept = 0). Each point is a patient in the study, and point colors indicate age group: <18 = cyan, 18-39 magenta, 40-59 grey, 60-80 black.

**Fig. 3:**
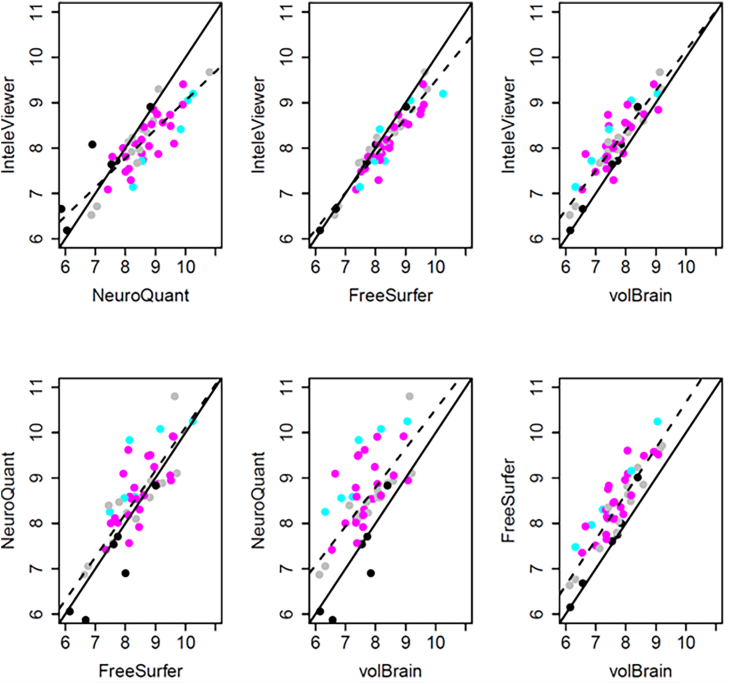
Illustrative relationships between hippocampal volumes (cm^3^) measured using different pairs of software programs. The best fit line is dashed (model information in Table 3, S1), and the reference line for perfect agreement is solid (slope = 1, intercept = 0). Each point represents a patient in the study, and point colors indicate age group: <18 = cyan, 18-39 magenta, 40-59 grey, 60-80 black.

Population-level statistics based on different tracing methods provides insights into the relative magnitude of bias involved. Software programs produced different estimated mean values for LVV, and the LVV estimated by manual tracing in InteleViewer® was the largest of all methods studied (Table 2). Of particular note, despite high consistency, mean LVV estimated in volBrain was 58% of the mean volume obtained from manual tracing. HV means varied less across software programs than LVV, with the smallest (volBrain) mean HV being 90% of the largest (NeuroQuant®). HV measured in volBrain was smaller than manual tracing, whereas NeuroQuant® and FreeSurfer® mean hippocampal volumes were larger than the mean HV from manual tracing. Upon further analysis, due to size-related bias (Fig. 2, Fig. 3), for many comparisons the means and standard deviations (SDs) do not sufficiently depict the differences between software programs.

**Table 2:** Mean and standard deviation (SD) for LVV and HV (cm^3^) by volumetric measurement program. InteleViewer® is the mean across raters per participant. Due to size-related measurement bias, descriptive statistics across programs should be interpreted with caution.

	Lateral Ventricle	Hippocampus
InteleViewer®	16.8 ± 6.0	8.1 ± 0.7
NeuroQuant®	15.7 ± 6.1	8.5 ± 1.0
FreeSurfer®	12.6 ± 5.2	8.3 ± 0.9
volBrain	9.8 ± 4.9	7.7 ± 0.8

The high agreement among software programs for LVV suggested that linear equations modeling relationships between each pair may lend insight into program-specific segmentation biases and allow conversion between software programs (Fig. 2; Table 3, S1). InteleViewer® manual tracing and NeuroQuant® did not have volume-biased measurements and required only addition of a small constant (1.38) to convert between them. FreeSurfer® and volBrain systematically underestimated LVV relative to NeuroQuant® and InteleViewer®. There were also volume-dependent biases associated with FreeSurfer® and volBrain, where larger LVVs were underestimated more than smaller volumes relative to InteleViewer® and NeuroQuant®. There was also a small volume-dependent bias between FreeSurfer® and volBrain. Visualization of age relative to measurements showed that biases were not confined to any particular age group (Fig. 2). The best-fit linear equation between Rater 1 and Rater 2 for LVV suggested that Rater 1 systematically overestimated LVV relative to Rater 2 for patients with the largest LVVs (Table S2, Fig. S2).

**Table 3:** Illustrative line equations relating LVV and HV measurements of mTBI patients from four software programs. Independent variables (x) in the left column, dependent variables (y) are in the top row. Coefficients are bold when the 95% confidence interval did not include zero for the intercept or one for the slope (full statistics in Table S1).

(x)	(y) = InteleViewer®	NeuroQuant®	FreeSurfer®	volBrain
**Lateral Ventricle**				
InteleViewer®		y = 0.99x-0.92	y = **0.85**x-**1.76**	y = **0.81**x-**3.72**
NeuroQuant®	y = 0.98x+**1.38**		y = **0.85**x-**0.88**	y = **0.80**x-**2.83**
FreeSurfer®	y = **1.14**x+**2.43**	y = **1.16**x+**1.16**		y = **0.95**x-**2.06**
volBrain	y = **1.20**x+**5.00**	y = **1.21**x+**3.83**	y = **1.05**x+**2.25**	
**Hippocampus**				
InteleViewer®		y = 1.13x-0.67	y = 1.10x-0.59	y = 0.92x+0.22
NeuroQuant®	y = **0.64x**+**2.67**		y = **0.72x**+**2.22**	y = **0.51x**+**3.37**
FreeSurfer®	y = **0.82x**+**1.26**	y = 0.95x+0.60		y = **0.79x**+**1.11**
volBrain	y = **0.88x**+**1.35**	y = 0.86x+1.91	y = 1.01x+0.57	

The HV measurements had lower consistency among software programs, casting doubt on whether equations could be used to convert measurements. NeuroQuant® had notably lower fidelity with the other software programs in measuring HV, as reflected in the smaller coefficients of determination (InteleViewer® r^2^ = 0.72, FreeSurfer® r^2^ = 0.68, volBrain r^2^ = 0.44; Table S1) and large spread of points in Fig. 3. Measurements of HV by InteleViewer®, FreeSurfer®, and volBrain were less similar than for LVV (r^2^ > 0.80), and there were volume-based biases for all pairs of software programs. Relative to InteleViewer®, measurements using FreeSurfer® were generally accurate for small volumes, but overestimated larger volumes, while volBrain underestimated HV overall, particularly for smaller volumes (Table 3, Fig. 3). Visualization of age relative to measurements suggested NeuroQuant® underestimated HV for participants aged 60-80 and overestimated HV for participants under 18 relative to volBrain (Fig. 3). The best-fit linear equations between pairs of human raters showed variability similar to that seen in comparisons among software programs, with intercepts between 0.58 and 3.62 and slopes from 0.60 to 1.01 (Table S2, Fig. S2).

## 4 Discussion

This study adds to the literature on relative consistency and agreement in measuring LVV and HV of mTBI patients with different software programs. The results suggest that automated software programs produce volumetric measurements that are generally consistent with each other and with manual programs. Results depend on the interaction between software programs and ROI, and the magnitude of bias is similar to that associated with different human raters. Most notable is the volume-based bias between software programs and manual tracing, suggesting that population means and SDs from different techniques should not be compared or simply offset to match each other. More importantly, a population measured in one type of software is not appropriate for calculating a z-score for a patient measured in different software. Longitudinal series with heterogeneous software are likely to produce a compromised trajectory. Complicating the problem, the biases were not consistent in type: for some combinations of ROI and software programs, numerical values between software programs were directly comparable, in others, data required numerical conversion to be directly comparable, and there were also ROI and software program combinations that were not sufficiently similar to allow comparison. Overall, LVV measurements were consistent enough among software programs to convert using linear equations. HV measurements were inconsistent and attempts to convert among software programs could add significant random noise to the data.

This study is consistent with others who report high inter-rater (between human raters) reliability when using manual tracing software programs, and the findings also reflect the generally appreciated difficulty of segmenting the hippocampus compared to the lateral ventricle (Akudjedu et al., 2018; Guenette et al., 2018). An inter-rater ICC of 0.91 was reported for ventricle volumes measured from computed tomography (CT) images using OsiriX manual tracing software (Toth et al., 2015). The inter-rater ICC in this study using InteleViewer® was higher, most likely due to the better resolution of MRI as compared to CT. Schmidt reported slightly lower inter-rater reliability based on manual tracing of the hippocampus compared to our results (Schmidt et al., 2018). Comparisons among software programs for measuring LVV and HV point to different conclusions and are therefore discussed separately.

### 4.1 Lateral ventricles

High consistency between automated and manual tracing programs for segmenting lateral ventricles has been previously reported. Kempton et al. compared ALVIN, FSL-FIRST, and FreeSurfer® and found excellent consistency (ICC≥0.941) (Kempton et al., 2011). Guenette et al. found excellent consistency between FreeSurfer® automated segmentation and neuroradiologist/neuroanatomist measurements for right and left lateral ventricles (ICC = 0.977, 0.964) (Guenette et al., 2018). A similar study comparing an automated method (ALVIN) to manual tracing of lateral ventricles from MRI in patients with Alzheimer's Disease and normal participants using MRI also reported excellent consistency (ICC = 0.973-0.996) (Ertekin et al., 2016). The findings from previous studies are also in accordance with our observation that age was not a significant contributor to agreement among tracing methods.

Nonetheless, the bias in mean LVV, particularly bias that became more pronounced as a function of size, suggests that LVV measurements should not be directly compared if estimated using different software programs. Underestimation of LVV by some automated software programs has been previously reported for NeuroQuant® compared to FreeSurfer®, with similarly high consistency (ICC>0.98) (Ochs et al., 2015; Ross et al., 2018). In contrast, an earlier study using FreeSurfer®, ALVIN, and FSL-First found overestimation of mean LVV in young adults, patients with Alzheimer's Disease, and infants, relative to manual tracing (Kempton et al., 2011). Kempton et al. did not investigate volume-dependent biases.

### 4.2 Hippocampus

Consistency between manual tracing and automated tracing software output was lower and more software-dependent for HV than for LVV. (Ross et al., 2018 reported consistency ICC = 0.86 for NeuroQuant® and FreeSurfer® for the hippocampus, slightly higher than in this study. Consistency in other studies has varied, with ICC for left and right hippocampi of 0.91, 0.92, respectively, in older adults in Zandifar et al. (2017) and 0.77, 0.79 in a population of older (mean age = 70.6) healthy controls in Shen et al. (2010).

Previous studies also disagree on the presence of an overall bias for HV measurements, and whether the bias is dependent on volume. A pediatric study of manual vs automated tracing found that FreeSurfer® overestimated volumes in both the hippocampus and amygdala (Schoemaker et al., 2016). Akudjedu et al. compared three automated volumetric programs to manual tracing and found overestimation of the hippocampus by the automated programs (Akudjedu et al., 2018). FreeSurfer® was found to overestimate HV compared to manual tracing in several studies (Morey et al., 2009; Schmidt et al., 2018; Shen et al., 2010; Zandifar et al., 2017), particularly for larger hippocampi in the older patients (median age = 75.5) in Zandifar et al. (2017). However, Cherbuin found no size-based bias in estimation of the hippocampus when comparing FreeSurfer® to manual segmentation in a sample of 403 adults aged 44-48 (Cherbuin et al., 2009) Measuring HV, Schmidt et al. reported a size-based bias in FreeSurfer® v5.3, but not FreeSurfer® 6.0 as used in this study, relative to manual segmentation (Schmidt et al., 2018). The inconsistent findings of automated relative to manual tracing may be associated with the training of the person doing the manual tracing, particularly how the boundaries between hippocampus and amygdala, entorhinal cortex, and parahippocampal gyrus are handled. Another possible explanation is that failing to remove problematic segmentation using automated methods can lead to lower reliability compared to manual tracing (Mulder et al., 2014).

The discrepancies between this study and others may be due to large variability in age from this study compared to theirs. Many of the studies that compare automated segmentation with manual tracing have been studying Alzheimer's Disease, so participants have been older adults. A previous study comparing FreeSurfer® segmentation of the hippocampus for younger (20-30 years) and older (60-70 years) adults reported that FreeSurfer® overestimated hippocampal volume overall in the younger group, while in the older group the overestimation was smaller, particularly for those with larger hippocampi (Wenger et al., 2014). The authors concluded that more caution must be exercised relative to patient age, since agreement ICC between FreeSurfer® and manual tracing for younger patients (aged 20-30) was 0.275, 0.381 for the left and right hippocampi, whereas for the older group (aged 60-70) ICC was 0.361, 0.577 (Wenger et al., 2014). In this study, the sample size was not sufficient to formally test for age effects, but visual inspection suggested that there may have been some cases where there was age-associated bias, such as NeuroQuant® HV for pediatric brains and for those above 60.

### 4.3 Comparative findings

The differences in results between LVV and HV may be due to size or the nature of their respective boundaries. Lateral ventricles have approximately twice the volume of the hippocampus. Koussis et al. compared NeuroQuant® and volBrain in 56 patients for eight regions of interest and found that regions with larger volumes had higher reliability than regions with smaller volumes (Koussis et al., 2023). Moreover, segmentation of the hippocampus, particular in differentiating it from the amygdala, is known to be difficult, e.g., (Zandifar et al., 2017). Previous studies of hippocampal segmentation described greater difficulties defining the boundary of the anterior-medial surface using FreeSurfer® (Morey et al., 2009) and variable problems in segmentation using NeuroQuant® and FreeSurfer®, with segmentation differing in quality even between the left and right sides of the same brain (Brinkmann et al., 2019). Automated and manually corrected volumes for the amygdala-hippocampal complex are more reproducible than for either structure alone (Guenette et al., 2018). A longitudinal study looking at within-person changes in hippocampal volume in patients with Alzheimer's Disease, mild cognitive impairment, or neither, reported that there was a visually incorrect segmentation for 7.5% of hippocampi that were segmented in FreeSurfer® (Mulder et al., 2014). User induced left-right bias termed ‘laterality of visual perception” bias can be as high as 11% in user-guided segmentation programs, based on a study in which left and right sides were switched using ‘mirror flipping/inverting’ (Maltbie et al., 2012). The effect was particularly strong in the hippocampus. Lateralized user bias may therefore degrade the performance of partially automated volumetric programs such as volBrain. In this study, the stability of the mean volumes among software programs and manual tracing, along with relatively small error estimates, masked the fact that reliability measures for HV, which consider within-person consistency and agreement, were considerably lower than for LVV, even without consideration of lateralized biases.

Another study which reported linear equations relating two software programs (Ross et al., 2018), NeuroQuant® and FreeSurfer®, used Bayesian regression to find the coefficients, rather than the linear regressions reported in this work. For the lateral ventricle the slope was steeper (i.e., 0.92 compared to 0.85) and the intercept more negative (−2.60 compared to −0.88) in Ross et al., with no overlap between the 95% confidence intervals of coefficients in this study and theirs. For the hippocampus, the slope was also steeper (1.12 compared to 0.72) and the intercept smaller (−1.32 compared to 2.22) in Ross et al. than in this study, with no overlap in the 95% confidence intervals. The linear equation relating manual tracing and FreeSurfer® reported in Morey et al. (2009) reflected a stronger volume bias compared to the results in this study. It is difficult to understand the reason for differences in the results using the same pair of software programs. It is likely that scanner technical effects and possibly patient age range played a role in the findings. The divergence between the coefficients suggests that even more caution is urged when attempting to translate findings between or within software programs, even for LVV.

This study has several limitations. All subjects had been diagnosed with mTBI. Patients with moderate and severe TBI often have lesions that challenge accurate segmentation (Diamond et al., 2020). Previous studies have found that patients with mTBI have abnormalities occurring at rates similar to those of normal controls (Patel et al., 2020). However, the burden of white matter hyperintensities was higher overall for mTBI patients compared to controls in the frontal cortex, which may make segmentation more difficult in this population compared to healthy controls (Karim et al., 2016). For practical reasons, there were some differences in the images used for manual ventricular (4 mm T2 slices) compared to automated tracing (1.2 mm T1 slices). Previous work on HV bias associated with slice thickness (1 mm to 5 mm) found no effect on mean volume or variance of resulting estimates (Laakso et al., 1997). Inter-method reliability (ICC) between NQ and FreeSurfer improved by 0.07 when using 1.0 mm compared to 1.2 mm slices in patients with mild cognitive impairment, with thicker slices tending to magnify differences between the two software programs (Yim et al., 2021). The comparatively thick slices used in this study may therefore have provided a conservative estimate of agreement between methods. Given the close agreement among all manual tracing methods for LVV, slice thickness did not appear to contribute in a major way to the findings. The time difference between performing such tracings on thinner slices is quite significant, limiting any potential clinically relevant application.

The small sample limited the analysis to volume-based bias, ignoring a formal analysis of potential age-based biases. Age-related biases need to be explored in a study with participants representing every life stage, as in this study. Another limitation is that the results of this study should not be generalized to different ROI in the brain because of the unknown importance of size and shape as a source of error. Previous studies have found that consistency ICCs for smaller brain regions with less well-defined borders, such as the pallidum and cerebellar white matter, were poor (Ochs et al., 2015). Thus, a trend can be identified that smaller regions with irregular borders may be associated with low ICC values, making generalization of findings specific to the hippocampus and lateral ventricle difficult.

In conclusion, LVV measurements in Inteleviewer®, FreeSurfer®, volBrain, and NeuroQuant® can reasonably be inter-converted in mTBI patients, given equations relevant to the machines and pulse sequences that were used. Although outside the scope of this study, harmonization across MRI machines, pulse sequences, and even drift in performance over time for the same scanner, is a problem that has not been fully addressed by existing approaches and is still a topic of active research (Abbasi et al., 2024). Bias associated with the scans likely interacts with the idiosyncrasies of volumetric segmentation approaches reported in this study to degrade the reproducibility of volumetric measurements. There would be substantial clinical benefits if direct conversion from one scan to another, given the details of MRI machine, patient position, pulse sequence, magnet strength, and software segmentation approach, were possible. HV measurements were inconsistent among software programs and among human raters, and attempts to convert between software programs would add significant random noise to the data. Clinicians and researchers should, however, be aware that conversion is necessary before data can be directly compared, even when working with population-level statistics. As deep learning models are being developed, the same systematic biases, particularly those that are size-dependent, are likely to arise. It is therefore critical to test results of new technologies against a known standard, such as manual tracing from multiple trained persons or well-understood automated tracing, before adopting new methods into the clinic.

## Disclaimer

This research was supported (in whole or in part) by HCA Healthcare and/or an HCA Healthcare affiliated entity. The views expressed in this publication represent those of the author(s) and do not necessarily represent the official views of HCA Healthcare or any of its affiliated entities.

## Research data for this article

The data used in this study are provided in Supplementary material, Tables S3 and S4.

## CRediT authorship contribution statement

**Lauren Carter:** Project administration, Supervision, Writing – original draft, Writing – review & editing. **Tapasya Surti:** Investigation, Writing – original draft, Writing – review & editing. **Tracy J. Abildskov:** Investigation, Methodology, Resources, Software, Writing – review & editing. **Eddie Shum:** Investigation, Writing – review & editing. **Amanda R. Hertzler:** Investigation, Writing – review & editing. **Meghana Dodda:** Investigation, Writing – review & editing. **Alan Rodriguez:** Methodology, Project administration, Software, Supervision, Validation, Writing – review & editing. **Colin Brown:** Investigation, Writing – review & editing. **Justin Roy Sanchez:** Investigation, Writing – review & editing. **Deen Tar:** Investigation, Writing – review & editing. **Leon Ma:** Investigation, Writing – review & editing. **Sean Beaty:** Investigation, Writing – review & editing. **Enrico Fazzini:** Conceptualization, Resources, Writing – review & editing. **Cheryl E. Hightower:** Conceptualization, Formal analysis, Project administration, Supervision, Validation, Visualization, Writing – original draft, Writing – review & editing. **Travis H. Snyder:** Conceptualization, Project administration, Resources, Software, Supervision, Writing – review & editing.

## Declaration of competing interest

The authors declare the following financial interests/personal relationships which may be considered as potential competing interests: TS owns interest in a start-up company which is currently developing software as a medical device that performs volumetric measurements from MRI data. No other authors have a potential competing interest.

## Data Availability

Data are in Supplementary Tables 3,4
