# Supplementary material for: Volume-based bias in automated measurements of lateral ventricle and hippocampal volumes of mild traumatic brain injury patients
Source: Neuroimage Rep. 2026 Jun 12;6(3):100361. doi: 10.1016/j.ynirp.2026.100361 (PMC13279000; doi:10.1016/j.ynirp.2026.100361)
Supplement: Multimedia component 1 [file mmc1.docx]

**Supplementary material**


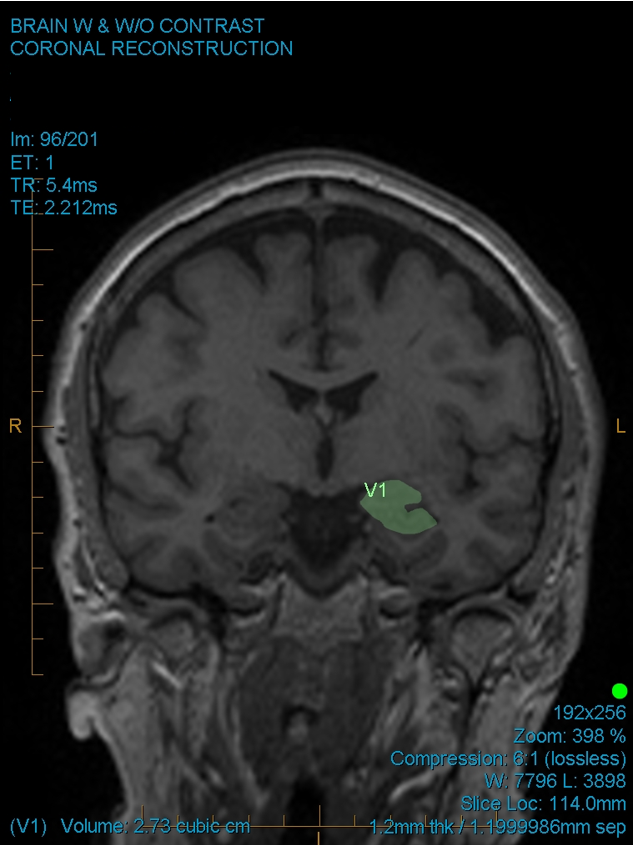


Figure S1. InteleViewer interface screenshot for the manual tracing of the left hippocampus from slice 96 or 201 from a coronal reconstructed image for a male, 51 years.


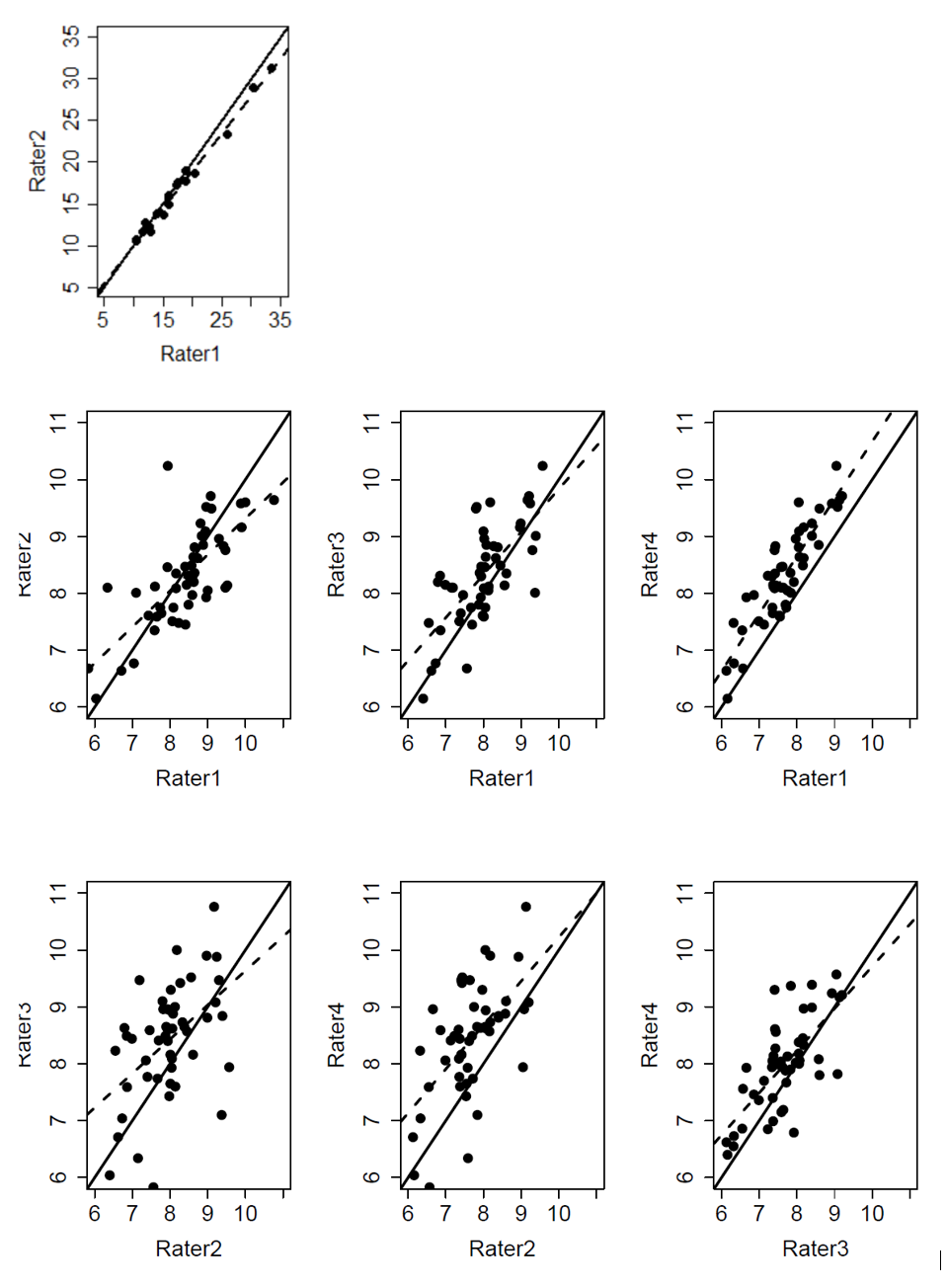


Figure S2. Illustrative relationships between lateral ventricle volumes (cm^3^; top row) and hippocampal volumes (cm^3^, lower two rows) measured manually in Inteleviewer by different human raters. The best fit line is dashed (model information in Table S2), and the reference line for perfect agreement is solid (slope=1, intercept=0).

**Table S1**. Illustrative lateral ventricle and hippocampus from mTBI patients’ regression results for pairs of software programs. Slopes and intercepts reported in line equations in Table 3, with this table adding the R-squared values and 95% confidence intervals for each pair of tracing software programs, are reported. Coefficients are bold when the 95% confidence interval did not include zero for the intercept or one for the slope. Line equations are in Table 3.

|  | | |  | **Lateral Ventricle** | | | **Hippocampus** | | | |
| --- | --- | --- | --- | --- | --- | --- | --- | --- | --- | --- |
| **Dependent variable (y)** | **Independent variable (x)** | **Intercept** | | | **Slope** | **r^2^** | **Intercept** | **Slope** | **r^2^** |  |
| InteleViewer® | NeuroQuant® | **1.38**  **0.23, 2.69** | | | 0.98  0.90, 1.03 | 0.97 | **2.67**  **1.66, 3.68** | **0.64**  **0.52, 0.75** | 0.72 |  |
| InteleViewer® | FreeSurfer® | **2.43**  **1.45, 3.53** | | | **1.14**  **1.07, 1.20** | 0.98 | **1.26**  **0.58, 2.06** | **0.82**  **0.73, 0.91** | 0.90 |  |
| InteleViewer® | volBrain | **5.00**  **3.82, 6.15** | | | **1.20**  **1.10, 1.29** | 0.97 | **1.35**  **0.41, 2.44** | **0.88**  **0.74, 1.00** | 0.81 |  |
| NeuroQuant® | InteleViewer® | -0.92  -2.11, 0.36 | | | 0.99  0.92, 1.04 | 0.97 | -0.67  -2.05, 1.03 | 1.13  0.92, 1.30 | 0.72 |  |
| NeuroQuant® | FreeSurfer® | **1.16**  **0.60, 1.87** | | | **1.16**  **1.12, 1.19** | 0.99 | 0.60  -0.88, 2.28 | 0.95  0.75, 1.13 | 0.68 |  |
| NeuroQuant® | volBrain | **3.83**  **2.87, 5.04** | | | **1.21**  **1.11, 1.28** | 0.98 | 1.91  -0.38, 4.36 | 0.86  0.56, 1.15 | 0.44 |  |
| FreeSurfer® | InteleViewer® | **-1.76**  **-2.62, -0.54** | | | **0.85**  **0.78, 0.89** | 0.98 | -0.59  -1.43, 0.24 | 1.10  0.99, 1.21 | 0.90 |  |
| FreeSurfer® | NeuroQuant® | **-0.88**  **-1.40, -0.35** | | | **0.85**  **0.82, 0.88** | 0.99 | **2.22**  **1.03, 3.50** | **0.72**  **0.56, 0.86** | 0.68 |  |
| FreeSurfer® | volBrain | **2.25**  **1.78, 2.70** | | | **1.05**  **1.01, 1.09** | 0.99 | 0.57  -0.50, 1.71 | 1.01  0.86, 1.15 | 0.80 |  |
| volBrain | InteleViewer® | **-3.72**  **-4.87, -2.35** | | | **0.81**  **0.73, 0.86** | 0.97 | 0.22  -0.75, 1.23 | 0.92  0.79, 1.04 | 0.81 |  |
| volBrain | NeuroQuant® | **-2.83**  **-3.74, -1.77** | | | **0.80**  **0.74, 0.84** | 0.98 | **3.37**  **2.15, 4.73** | **0.51**  **0.34, 0.65** | 0.44 |  |
| volBrain | FreeSurfer® | **-2.06**  **-2.55, -1.54** | | | **0.95**  **0.90, 0.98** | 0.99 | **1.11**  **0.18, 1.90** | **0.79**  **0.70, 0.90** | 0.80 |  |

**Table S2**. Illustrative lateral ventricle volume (LVV, 2 raters) and hippocampal volume (HV, 4 raters) from mTBI patients’ regression results for pairs of human raters. Slopes and intercepts are reported with 95% bootstrapped confidence intervals and R-squared values for each pair of manual reviewers, are reported. Coefficients are bold when the 95% confidence interval did not include zero for the intercept or one for the slope. Related scatterplots appear in Figure S2. Means from all raters are used to represent manual tracing in comparisons with automated software.

|  | Rater | Rater | Intercept | slope | r^2^ |
| --- | --- | --- | --- | --- | --- |
| LVV | 1 | 2 | **1.11**  **(0.33, 2.06)** | **0.90**  **(0.83, 0.94)** | 0.99 |
| HV | 1 | 2 | **2.96**  **(1.55, 4.46)** | **0.64**  **(0.46, 0.80)** | 0.54 |
|  | 1 | 3 | **2.30**  **(0.47, 4.20)** | **0.75**  **(0.51, 0.98)** | 0.50 |
|  | 1 | 4 | 0.58  (-0.47, 1.67) | 1.01  (0.87, 1.14) | 0.80 |
|  | 2 | 3 | **3.62**  **(0.56, 6.58)** | **0.60**  **(0.23, 0.99)** | 0.24 |
|  | 2 | 4 | **2.50**  **(0.10, 5.27)** | 0.77  (0.42, 1.08) | 0.35 |
|  | 3 | 4 | **2.30**  **(0.99, 3.93)** | **0.74**  **(0.52, 0.92)** | 0.49 |

**Table S3**. Raw data for total hippocampal volumes (cm^3^).

| age | sex | Intracranial volume | Mean Inteleviewer Total | Freesurfer Total | NQ Total | Neuroreader Total | volbrain Total | Total:Manual Reader 1 | Total:Manual Reader 2 | Total:Manual Reader 3 | Total:Manual Reader 4 |
| --- | --- | --- | --- | --- | --- | --- | --- | --- | --- | --- | --- |
| 19 | F | 1453.4 | 7.9 | 8.2 | 8.5 | 6.8 | 7.9 | 8.2 | 8.6 | 6.8 | 7.9 |
| 40 | F | 1395.5 | 7.5 | 7.5 | 8.0 | 7.4 | 7.0 | 7.5 | 8.1 | 7.4 | 7.0 |
| 75 | M | 1779.2 | 8.1 | 8.0 | 6.9 | 9.4 | 7.8 | 8.0 | 7.1 | 9.4 | 7.8 |
| 56 | M | 1803.0 | 9.7 | 9.6 | 10.8 | 9.2 | 9.1 | 9.6 | 10.8 | 9.2 | 9.1 |
| 29 | M | 1666.0 | 8.1 | 8.5 | 8.3 | 7.9 | 7.6 | 8.5 | 8.4 | 7.9 | 7.6 |
| 20 | F | 1427.6 | 7.1 | 7.4 | 7.4 | 6.9 | 6.5 | 7.4 | 7.6 | 6.9 | 6.6 |
| 13 | F | 1289.8 | 7.1 | 7.5 | 8.3 | 6.5 | 6.3 | 7.5 | 8.2 | 6.6 | 6.3 |
| 14 | F | 1529.8 | 7.7 | 8.3 | 8.6 | 6.9 | 7.2 | 8.3 | 8.5 | 6.9 | 7.2 |
| 56 | M | 1716.2 | 8.6 | 8.9 | 8.9 | 8.1 | 8.6 | 8.9 | 8.9 | 8.1 | 8.6 |
| 32 | M | 1721.3 | 8.5 | 9.1 | 8.9 | 8.0 | 8.1 | 9.1 | 8.9 | 8.0 | 8.1 |
| 62 | M | 1336.7 | 7.7 | 7.7 | 7.7 | 7.7 | 7.7 | 7.8 | 7.7 | 7.7 | 7.7 |
| 55 | F | 1352.9 | 8.9 | 9.2 | 8.9 | 9.0 | 8.4 | 9.2 | 8.8 | 9.0 | 8.4 |
| 29 | F | 1449.0 | 8.5 | 8.8 | 9.5 | 8.3 | 7.4 | 8.8 | 9.4 | 8.3 | 7.4 |
| 32 | F | 1571.5 | 7.7 | 8.1 | 8.6 | 7.0 | 7.4 | 8.2 | 8.4 | 7.0 | 7.4 |
| 35 | F | 1549.5 | 8.1 | 8.1 | 9.6 | 7.2 | 7.6 | 8.1 | 9.5 | 7.2 | 7.6 |
| 18 | F | 1524.0 | 9.1 | 9.2 | 10.1 | 9.0 | 8.2 | 9.2 | 9.9 | 9.0 | 8.2 |
| 26 | F | 1430.0 | 8.0 | 8.3 | 8.8 | 7.9 | 7.3 | 8.3 | 8.6 | 7.9 | 7.3 |
| 46 | M | 1550.2 | 9.3 | 9.7 | 9.1 | 9.2 | 9.2 | 9.7 | 9.1 | 9.2 | 9.2 |
| 60 | F | 1284.3 | 6.5 | 6.6 | 6.9 | 6.6 | 6.1 | 6.6 | 6.7 | 6.6 | 6.1 |
| 49 | M | 1598.1 | 7.7 | 7.6 | 7.6 | 8.0 | 7.6 | 7.6 | 7.7 | 8.0 | 7.6 |
| 23 | M | 1639.6 | 8.8 | 9.5 | 8.9 | 7.8 | 9.1 | 9.5 | 9.0 | 7.8 | 9.1 |
| 18 | F | 1451.7 | 8.4 | 8.1 | 9.8 | 8.6 | 7.4 | 8.1 | 9.5 | 8.6 | 7.4 |
| 57 | F | 1512.2 | 8.5 | 8.8 | 8.6 | 8.4 | 8.0 | 8.8 | 8.7 | 8.4 | 8.1 |
| 51 | F | 1482.2 | 8.4 | 8.5 | 8.6 | 8.4 | 8.2 | 8.5 | 8.6 | 8.5 | 8.2 |
| 62 | F | 1357.1 | 7.6 | 7.6 | 7.5 | 8.0 | 7.5 | 7.6 | 7.4 | 8.0 | 7.5 |
| 27 | M | 1733.4 | 8.7 | 9.5 | 9.1 | 7.8 | 8.6 | 9.5 | 9.1 | 7.8 | 8.6 |
| 12 | F | 1577.9 | 7.7 | 8.0 | 8.6 | 7.5 | 6.9 | 8.0 | 8.6 | 7.5 | 6.9 |
| 57 | F | 1445.6 | 8.3 | 8.6 | 8.7 | 8.1 | 8.1 | 8.6 | 8.6 | 8.1 | 8.1 |
| 26 | F | 1443.2 | 7.5 | 7.7 | 8.1 | 7.4 | 7.4 | 7.7 | 7.8 | 7.4 | 7.4 |
| 36 | M | 1654.3 | 9.4 | 9.6 | 9.9 | 9.2 | 8.9 | 9.6 | 9.9 | 9.2 | 8.9 |
| 59 | M | 1516.6 | 8.1 | 8.4 | 8.1 | 8.6 | 7.4 | 8.4 | 8.2 | 8.6 | 7.4 |
| 46 | F | 1316.6 | 6.7 | 6.8 | 7.1 | 6.7 | 6.3 | 6.8 | 7.0 | 6.7 | 6.3 |
| 25 | M | 1469.0 | 7.9 | 7.9 | 9.1 | 7.9 | 6.7 | 7.9 | 9.0 | 7.9 | 6.7 |
| 49 | F | 1495.3 | 7.9 | 8.1 | 8.2 | 8.0 | 7.4 | 8.1 | 8.2 | 8.0 | 7.4 |
| 38 | F | 1375.1 | 8.2 | 8.4 | 8.5 | 7.9 | 7.8 | 8.4 | 8.7 | 7.9 | 7.8 |
| 77 | F | 1221.1 | 6.7 | 6.7 | 5.9 | 7.6 | 6.6 | 6.7 | 5.8 | 7.6 | 6.6 |
| 31 | M | 1651.3 | 8.7 | 8.8 | 9.5 | 9.3 | 7.4 | 8.8 | 9.5 | 9.3 | 7.4 |
| 33 | M | 1802.7 | 9.0 | 9.6 | 9.9 | 8.2 | 8.1 | 9.6 | 10.0 | 8.2 | 8.1 |
| 22 | M | 1635.0 | 8.6 | 9.0 | 9.2 | 8.0 | 8.0 | 9.0 | 9.3 | 8.0 | 8.0 |
| 43 | M | 1571.8 | 7.7 | 7.4 | 8.4 | 7.7 | 7.1 | 7.5 | 8.4 | 7.7 | 7.1 |
| 46 | M | 1488.4 | 8.0 | 7.8 | 8.5 | 7.9 | 7.7 | 7.8 | 8.5 | 7.9 | 7.7 |
| 70 | F | 1186.8 | 6.2 | 6.2 | 6.1 | 6.4 | 6.2 | 6.2 | 6.0 | 6.4 | 6.2 |
| 36 | F | 1377.1 | 8.5 | 8.6 | 8.6 | 8.3 | 8.2 | 8.6 | 8.7 | 8.3 | 8.2 |
| 41 | F | 1350.3 | 8.2 | 8.0 | 8.2 | 8.1 | 7.8 | 8.1 | 9.0 | 8.1 | 7.8 |
| 35 | F | 1284.1 | 7.8 | 7.8 | 8.0 | 8.0 | 7.3 | 7.8 | 8.1 | 8.1 | 7.4 |
| 39 | F | 1524.3 | 8.0 | 8.5 | 7.9 | 8.0 | 7.6 | 8.5 | 7.9 | 8.0 | 7.6 |
| 22 | M | 1463.6 | 7.8 | 8.1 | 7.6 | 8.1 | 7.4 | 8.1 | 7.6 | 8.1 | 7.4 |
| 64 | M | 1766.5 | 8.9 | 9.0 | 8.8 | 9.4 | 8.4 | 9.0 | 8.8 | 9.4 | 8.4 |
| 18 | M | 1670.0 | 9.2 | 10.2 | 10.3 | 9.6 | 9.1 | 10.2 | 7.9 | 9.6 | 9.1 |
| 28 | F | 1370.9 | 7.3 | 8.1 | 8.2 | 7.1 | 7.6 | 8.1 | 6.3 | 7.2 | 7.6 |

**Table S4**. Raw data for total lateral ventricle volumes (cm^3^).

| age | sex | Intracranial volume | Manual tracing (InteleViewer) Mean | NeuroQuant (3.0) | FreeSurfer V6 | VolBrain | InteleViewer (Reader 1) | InteleViewer (Reader 2) |
| --- | --- | --- | --- | --- | --- | --- | --- | --- |
| 29 | M | 1594.2 | 17.3 | 16.4 | 13.1 | 10.0 | 17.3 | 17.3 |
| 31 | M | 1623.2 | 24.6 | 23.2 | 19.2 | 15.6 | 23.3 | 25.8 |
| 63 | F | 1614.1 | 16.1 | 16.3 | 11.7 | 8.2 | 16.1 | 16.0 |
| 35 | M | 1627.5 | 11.6 | 11.5 | 7.9 | 4.5 | 11.6 | 11.7 |
| 18 | F | 1466.2 | 13.9 | 12.0 | 9.7 | 7.2 | 13.8 | 14.1 |
| 18 | F | 1570.3 | 12.5 | 10.7 | 8.3 | 5.7 | 12.2 | 12.7 |
| 17 | F | 1562.7 | 11.8 | 12.2 | 9.3 | 6.6 | 11.8 | 11.8 |
| 37 | M | 1577.8 | 17.6 | 15.4 | 11.9 | 9.6 | 17.6 | 17.5 |
| 32 | F | 1399.2 | 19.5 | 17.7 | 7.2 | 5.2 | 18.6 | 20.4 |
| 56 | F | 1321.2 | 10.6 | 9.1 | 14.2 | 11.7 | 10.6 | 10.5 |
| 38 | F | 1400.7 | 15.5 | 12.3 | 10.4 | 8.1 | 14.9 | 16.0 |
| 41 | F | 1546.7 | 12.2 | 10.5 | 8.5 | 6.4 | 11.6 | 12.9 |
| 35 | F | 1327.5 | 18.3 | 17.5 | 14.2 | 11.6 | 17.7 | 18.8 |
| 36 | F | 1418.4 | 14.4 | 11.9 | 9.1 | 6.3 | 13.6 | 15.2 |
| 64 | M | 1532.0 | 29.6 | 28.8 | 23.8 | 20.8 | 28.9 | 30.3 |
| 51 | M | 1717.4 | 32.4 | 31.4 | 26.0 | 22.6 | 31.3 | 33.5 |
| 28 | F | 1445.2 | 12.4 | 11.9 | 9.4 | 6.7 | 12.7 | 12.1 |
| 27 | M | 1611.2 | 18.9 | 19.1 | 15.4 | 12.5 | 18.9 | 18.9 |
| 41 | F | 1527.8 | 10.6 | 11.0 | 8.7 | 6.6 | 10.7 | 10.4 |
| 46 | F | 1359.4 | 15.9 | 15.8 | 13.2 | 10.4 | 15.9 | 15.9 |
